# Modification of the classical Lancefield assay of group A streptococcal killing to reduce inter-donor variation

**DOI:** 10.1016/j.mimet.2016.03.015

**Published:** 2016-05

**Authors:** Mark Reglinski, Nicola N. Lynskey, Shiranee Sriskandan

**Affiliations:** Faculty of Medicine, Imperial College London, Hammersmith Campus, Du Cane Road, London W12 0NN, United Kingdom

**Keywords:** *Streptococcus pyogenes*, Group A streptococcus, Lancefield assay, Surrogate of immunity

## Abstract

The lack of a surrogate-of-immunity assay presents a major barrier to *Streptococcus pyogenes* research. Modification of the Lancefield assay to include an antibody digestion step reduced inter-donor variation and permitted detection of the anti-streptococcal activity of intravenous immunoglobulin and convalescent serum, thus facilitating retrospective evaluation of immunity using stored samples.

## Highlights

• The lack of a surrogate-of-immunity assay is a major barrier to *S. pyogenes* research.
• Pre-treatment of whole blood with IdeS standardised bacterial growth between donors.
• IdeS was subsequently inhibited using a non-cytotoxic iodoacetamide derivative.
• The bactericidal action of stored samples could then be measured by Lancefield assay.
• The modified assay facilitates retrospective dissection of anti-*S. pyogenes* immunity.

The generation of anti-streptococcal antibody during *Streptococcus pyogenes* infection is known to afford serotype specific protection against secondary infection, and to prevent growth of the organism in the blood of convalescent donors (Lancefield, 1959). Inoculation of a known quantity of *S. pyogenes* into donor whole blood and measurement of the degree of multiplication over 3 h is a well-established *in vitro* assay for *S. pyogenes* immunity that was first described by Edgar Todd in 1927 (Todd, 1927) and immortalised by Rebecca Lancefield over subsequent decades (Lancefield, 1959). More recently, the Lancefield assay has been modified for the screening of vaccine antigens through supplementation of whole blood from healthy donors with serum from vaccinated animals or humans (Caro-Aguilar et al., 2013, Hu et al., 2002, Kotloff et al., 2004). A major barrier to use of the Lancefield assay is the frequent existence of natural immunity to *S. pyogenes* within the donor blood utilised. This necessitates the use of blood from a significant number of human donors, and/or pre-screening for endogenous anti-streptococcal immunity prior to the assay being conducted (Moreland et al., 2014, Bauer et al., 2012).

The Immunoglobulin G-degrading enzyme of *S. pyogenes* (IdeS) is a secreted cysteine protease that cleaves human immunoglobulin (Ig)G at the hinge region, preventing FcR recognition and phagocytic ingestion of opsonised bacteria (von Pawel-Rammingen et al., 2002). The enzyme promotes complete digestion of IgG in whole blood in a matter of minutes (Johansson et al., 2008), raising the possibility that it could be exploited to facilitate inactivation of the endogenous anti-streptococcal antibodies that prevent the use of some donor whole blood samples in Lancefield assays. However, downstream detection of antibody in samples from convalescent patients and/or vaccinated subjects would require the enzyme to be inhibited following endogenous antibody digestion. Alkylating agents such as iodoacetamide can inhibit IdeS-mediated IgG cleavage (von Pawel-Rammingen et al., 2002) but are too cytotoxic for use in a whole blood assay. As a solution, we hypothesised that the commercially available, membrane-impermeable iodoacetamide derivative 4-Acetamido-4′-((iodoacetyl)amino)Stilbene-2,2′-Disulfonic Acid (A484, Molecular Probes) would inhibit IdeS without promoting intracellular alkylation; and thus allow the anti-streptococcal activity of exogenously applied samples to be evaluated (Johnson, 2010).

**Fig. 1:**
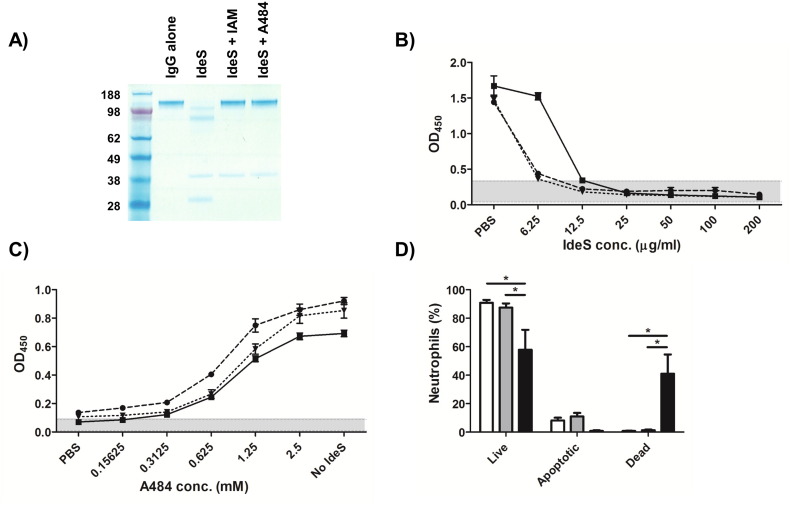
**Establishing the modified Lancefield assay conditions**. A) IdeS is inhibited by A484. 2 μg of IVIG (all lanes) was incubated with 0.5 μg of IdeS in presence of 10 mM iodoacetamide (IAM), 10 mM A484 or PBS. Reactions were visualised by SDS-PAGE under non-reducing conditions. Molecular mass markers are given in kilodaltons. B and C) IdeS can reduce the anti-streptococcal activity of whole blood but not in the presence of A484. Plasma from B) IdeS treated or C) IdeS/A484 treated blood samples (n = 3) were evaluated for anti-streptococcal activity by ELISA. Grey shading represents the upper and lower OD_450_ values from wells incubated with PBS in place of plasma. Data: mean ± SD. D) Evaluation of A484 impact on human neutrophils. Donor neutrophils (n = 3) were incubated with PBS (white bars), 2.5 mM A484 (grey bars) or 2.5 mM iodoacetamide (black bars) for 4 h, and the percentage of live, apoptotic and dead neutrophils was determined by flow cytometry following FITC-annexin V/PI staining. Data: mean ± SD. *p < 0.05, two-way ANOVA with Bonferroni's correction.

We first confirmed that A484 was capable of inhibiting the antibody-cleaving activity of IdeS using the clinical pooled plasma product intravenous immunoglobulin (IVIG) as a source of purified immunoglobulin. In 10 mM Tris–HCl (pH 8), 10 mM A484 was shown to effectively inhibit 0.5 μg of IdeS, and prevent the otherwise complete digestion of 2 μg of IVIG in 1 h at 37 °C (Fig. 1A). The ability of IdeS to digest the endogenous anti-streptococcal antibodies present within heparinised donor whole blood was next determined by co-incubation of whole blood, from an approved sub-collection of the Imperial College Tissue Bank, with IdeS (6.25–200 μg/ml) or PBS for 1 h at 37 °C. Following IdeS treatment, heat inactivated plasma (from each whole blood sample) was diluted 1:100, and anti-streptococcal antibody activities were measured by ELISA using plates coated with overnight M1 *S. pyogenes* cultures (strain H305) diluted to an OD_600_ of 0.5 in PBS as previously described (Reglinski et al., 2016). Bound, intact antibodies were detected using a 1:20,000 dilution of Fc-specific, HRP-conjugated goat anti-human IgG. An IdeS concentration of 25 μg/ml was shown to reduce the anti-*S.* *pyogenes* activity of the whole blood samples to below the limit of detection (Fig. 1B).

To determine if the antibody-cleaving activity of IdeS could be inhibited by A484 in whole blood, blood samples were co-incubated with 25 μg/ml IdeS and A484 (2.5 mM–0.15625 mM) for 1 h at 37 °C, and the ELISA repeated. IdeS activity was inhibited by 2.5 mM of A484 (Fig. 1C). To ensure that A484 would not cause neutrophil death, freshly isolated human neutrophils (purified from blood using MACSxpress® Neutrophil Isolation Kit, Miltenyi Biotec) in PBS supplemented with 10% foetal bovine serum, were coincubated with A484 and then stained using FITC-Annexin V Apoptosis Detection Kit I (BD Pharmingen). In contrast to 2.5 mM iodoacetamide, 2.5 mM of A484 did not induce cell death over the 4 h period required to complete the assay (Fig. 1D). Together these data provided the necessary conditions for IdeS-mediated ablation of endogenous anti-streptococcal antibody from donor whole blood, and subsequent inhibition of IdeS with the non-cytotoxic iodoacetamide derivative A484.

**Fig. 2:**
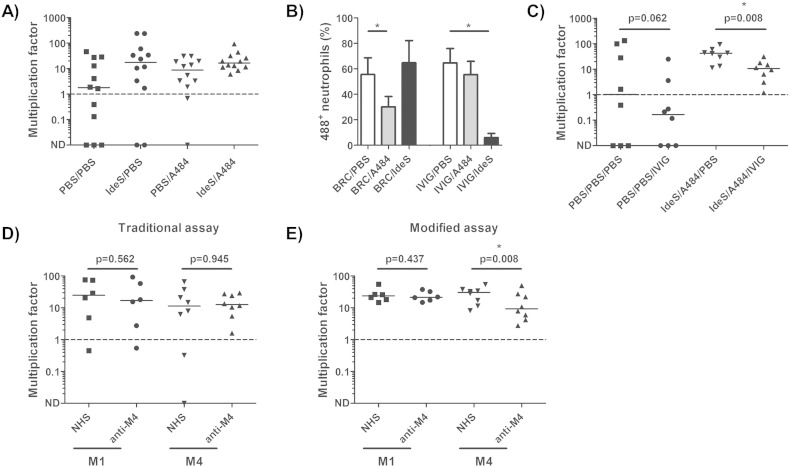
**IdeS**/**A484 modification of Lancefield assay**. A) Sequential addition of IdeS and A484 renders immune donor blood susceptible to streptococcal growth. Donor blood samples (n = 12) were pre-treated with IdeS and/or A484 and supplemented with 20–200 CFU of *S. pyogenes* (serotype M1 strain H305). Net bacterial growth (dashed line) was defined by a multiplication factor > 1. B) A484 inhibits complement but not Ig-mediated opsonophagocytosis. Donor neutrophils (n = 3) were incubated with baby rabbit complement (BRC) or IVIG in the presence of PBS (white bars), 2.5 mM A484 (grey bars) or 25 μg/ml IdeS (black bars) prior to the addition of 488^+^ GAS cells. The percentage of 488^+^ neutrophils was determined by flow cytometry. *p < 0.05, two-tailed student's t-test. C) IdeS/A484 treatment of donor whole blood (n = 8) permits detection of the anti-streptococcal activity of IVIG. *p < 0.05, Wilcoxon matched-pairs signed rank test. D and E) Effect of 10% normal human serum (NHS) or convalescent antiserum from M4-infected patient (anti-M4) on growth of strain H305 (M1, n = 6) or strain H914 (M4, n = 8) in a D) traditional Lancefield assay or E) modified Lancefield assay (IdeS/A484 pre-treatment of whole blood). *p < 0.05, Wilcoxon matched-pairs signed rank test.

Having outlined the necessary conditions for selected endogenous antibody digestion, Lancefield assays were performed using 270 μl of whole blood from twelve different donors, and modified by addition of 25 μg/ml of IdeS for 1 h, followed by 2.5 mM of A484 for 30 min. To prepare the bacterial inoculum, overnight *S. pyogenes* (M1, strain H305) cultures were inoculated 1:10 into fresh Todd-Hewitt broth and incubated at 37 °C with 5% CO_2_ to an OD_600_ of 0.15–0.3. Cultures were then diluted 1:5000 in PBS, to provide an inoculum of approximately 20–200 CFU in 15 μl, and plated to allow precise quantification of the starting inoculum. 15 μl of bacterial suspension was inoculated into IdeS/A484-treated or sham-treated whole blood reactions, incubated for 3 h, then plated out in triplicate to allow enumeration of bacterial survival (CFU ml^− 1^_(t_ _=_ _3_ _h)_/CFU ml^− 1^_(t_ _=_ _0_ _h)_). Compared with sham-treated controls, IdeS/A484 treatment facilitated survival and growth of *S. pyogenes* even in samples that had otherwise demonstrated endogenous bactericidal activity as a result of pre-existing immunity. More importantly, the reagents substantially reduced the observed variation in *S. pyogenes* multiplication obtained from the twelve donors (Fig. 2A).

It was noted that the addition of A484 appeared to decrease the bactericidal activity of donor samples, suggesting that the reagent may impact on opsonophagocytic uptake; notwithstanding the lack of any effect on neutrophil apoptosis. To explore this observation, purified human neutrophils were adjusted to 2 × 10^6^ cells/ml, pre-treated with 2.5 mM of A484 in the presence of IVIG or baby rabbit complement, and co-incubated with Oregon Green 488-X, succinimidyl ester 6-isomer (Molecular Probes) labelled GAS cells at a ratio of 10:1. After a 30 min co-incubation the percentage of neutrophils containing labelled bacteria in the baby rabbit serum reactions was reduced by approximately 30% in the presence of A484. In contrast, antibody mediated uptake was unaffected by the presence of A484, suggesting that the reagent may be specifically impacting on complement mediated uptake (Fig. 2B).

We next determined whether it was possible to detect anti-*S. pyogenes* antibody activity in exogenous IVIG (2 mg/ml) when added to the modified Lancefield whole blood reactions (Fig. 2C). IVIG was able to significantly limit M1 *S. pyogenes* growth, and, through application of the modified Lancefield assay, this effect was apparent in all donor samples. Finally we evaluated whether it was possible to detect anti-*S. pyogenes* activity in convalescent serum obtained from a patient 21 d following recovery from invasive M4 *S. pyogenes* infection. Although no increase in bacterial multiplication was observed upon IdeS/A484 pre-treatment, the inter-donor variation was reduced sufficiently to demonstrate that convalescent patient serum, but not normal human serum, was capable of promoting serotype specific killing of the M4 strain H914, an invasive clinical isolate recovered from the blood of the patient at the time of acute infection. In contrast, the same convalescent serum sample was unable to limit growth of a serotype M1 isolate, highlighting both the serotype specificity of the antibody response to *S. pyogenes*, and the potential applicability of the described protocol as an *in vitro* surrogate-of-immunity assay (Fig. 2D and E).

In conclusion, pre-treatment of donor whole blood with the IgG degrading enzyme IdeS successfully reduced the inter-donor variation associated with the Lancefield assay, and rendered immune whole blood susceptible to *S. pyogenes* multiplication. Notwithstanding the non-specific impact of A484 on complement mediated opsonophagocytosis, the modified assay was capable of detecting serotype specific killing using stored patient serum, thus facilitating retrospective dissection of the bactericidal antibody response to *S. pyogenes*. While the method will require further evaluation using a wider range of paired strains and serum samples, it may prove beneficial for determining the immune status of populations during *S. pyogenes* outbreaks, or, more importantly as a surrogate of immunity during vaccine trials.
